# Treatment and survival of *de novo* HER2-positive metastatic breast cancer across regional cancer networks in the Netherlands: a cohort study

**DOI:** 10.1016/j.esmorw.2026.100722

**Published:** 2026-08-24

**Authors:** N. Ding, S.M.E. Geurts, K.E.P.E. Hermans, I.J.H. Vriens, R. Visserman, L.F.S. Kooreman, N.A.J.B. Peters, E.M. Heuts, J. Verloop, A.C. Voogd, V.C.G. Tjan-Heijnen

**Affiliations:** 1 Division of Medical Oncology, Department of Internal Medicine, GROW Research Institute for Oncology and Reproduction, Maastricht University Medical Centre, Maastricht, the Netherlands; 2 Department of Pathology, GROW Research Institute for Oncology and Reproduction, Maastricht University Medical Centre, Maastricht, the Netherlands; 3 Department of Internal Medicine, Sint Jans Gasthuis Hospital, Weert, the Netherlands; 4 Department of Surgery, Maastricht University Medical Centre, Maastricht, the Netherlands; 5 Department of Research and Development, Netherlands Comprehensive Cancer Organisation, Utrecht, the Netherlands; 6 Department of Epidemiology, Maastricht University, Maastricht, the Netherlands

**Keywords:** breast neoplasms, ErbB-2, health care disparities, neoplasm metastasis, receptor, survival

## Abstract

**Background:**

Human epidermal growth factor receptor 2 (HER2)-targeted therapy has improved survival in HER2-positive metastatic breast cancer (MBC). Real-world practice may differ across oncologic networks, even within countries with uniform treatment guidelines. We assessed regional variation in first-line treatment and overall survival (OS) among patients with *de novo* HER2-positive MBC in the Netherlands in 2013-2023.

**Patients and methods:**

Data from the Netherlands Cancer Registry were analysed. The registry includes seven regions, anonymised as A-G. Crude regional proportions of first-line HER2-targeted therapy were described. Inverse probability of treatment weighting (IPTW) was applied, with region as the exposure. Risk ratios for receiving HER2-targeted therapy were estimated using IPTW-adjusted Poisson regression. OS was analysed using the Kaplan–Meier method and IPTW-adjusted Cox regression, additionally adjusting for baseline covariates and first-line treatment.

**Results:**

Among 2158 patients, use of first-line HER2-targeted therapy ranged from 70% (region F) to 88% (region C). After adjustment, no regional differences were observed (*P* ≥ 0.05), except for patients in region C, who were more likely to receive HER2-targeted therapy versus those in region A (risk ratio 1.09, 95% confidence interval, 1.02-1.16, *P* = 0.01). Observed median OS ranged from 42.8 months (region A) to 74.9 months (region B). After adjustment, no statistically significant regional differences in OS were observed. Regions B and E showed borderline longer OS versus region A (hazard ratio 0.77 and 0.77, *P* = 0.06 and 0.07, respectively).

**Conclusions:**

No large regional differences in first-line HER2-targeted therapy were observed among *de novo* HER2-positive MBC patients in the Netherlands. The observed regional OS difference could largely be explained by differences in baseline patient characteristics.

## Highlights

• Crude variation in first-line HER2-targeted therapy existed across Dutch regions.
• After IPTW adjustment, no large regional difference in first-line therapy remained.
• Regional survival variation diminished after adjustment for case-mix factors.
• Treatment access and OS were comparable across Dutch regions.
• Detailed data are needed to properly assess quality of delivered care in real life.

## Introduction

Approximately 15%-20% of breast cancers are human epidermal growth factor receptor 2 (HER2)-positive, a subtype historically associated with aggressive disease and poor prognosis.^1^ Among these patients, 15%-50% develop metastatic recurrence after treatment with curative intent, while 3%-10% present with metastatic disease at initial diagnosis, that is, *de novo* metastatic breast cancer (MBC).^1^^,^^2^ The advent of HER2-targeted therapies, such as trastuzumab and pertuzumab, has markedly improved outcomes of these patients.^3^, ^4^, ^5^ In the metastatic setting, median overall survival (OS) now approaches 5 years, and approximately one in five patients survives beyond 8 years after diagnosis.^6^^,^^7^

Despite uniform guideline recommendations, real-world use of HER2-targeted therapy differs across patients, countries, and health care systems.^8^^,^^9^ Studies from the Netherlands, France, and England have reported differences in access to HER2-targeted agents, often related to socioeconomic factors, hospital type (e.g. academic versus non-academic), and regional organisation of care (e.g. variation in concentration of specialised breast cancer centres or access to multidisciplinary tumour boards).^10^, ^11^, ^12^ In the Netherlands, cancer care is standardised mainly within national oncology networks, aimed at ensuring equal access.^13^ These networks support multidisciplinary collaboration and the implementation of guidelines. However, differences in referral pathways and local decision making can still lead to variation in care despite national guidelines.^14^ Unfortunately, data on within-country regional variation in the treatment and survival of patients with *de novo* HER2-positive MBC remain sparse.

This study aimed to evaluate regional variation in the use of first-line treatment and OS among patients with *de novo* HER2-positive MBC in the Netherlands between 2013 and 2023. Using population-based data from the Netherlands Cancer Registry (NCR), we assessed whether treatment patterns and outcomes differed across regions after the nationwide implementation of pertuzumab-based first-line therapy. Demonstrating the presence and understanding the causes of regional differences may help reduce potential inequalities in access to optimal care for this patient group.

## Patients and methods

### Study objectives

The primary objective was to examine regional variation in first-line treatment choice among patients diagnosed with *de novo* HER2-positive MBC in the Netherlands between 2013 and 2023. The secondary objective was to assess potential differences in OS across regions.

### Data collection

Data were obtained from the NCR, which records detailed clinical and treatment information for all patients newly diagnosed with cancer in the Netherlands. Trained data managers extract information directly from medical records using uniform national coding guidelines. Recorded variables include age, sex, clinical tumour and nodal stage, histology, tumour grade, hormone receptor status, and treatments administered within the first 9-12 months after diagnosis. Vital status is updated annually through linkage with the Municipal Personal Records Database. The date of this linkage was used as the date of last follow-up. As recurrent breast cancer is not systematically registered in the NCR, this analysis was restricted to patients with *de novo* HER2-positive MBC.

### Study population

Patients were included if they were diagnosed with *de novo* HER2-positive MBC between 1 January 2013 and 31 December 2023. Vital status was updated in March 2025. According to the regulations of the Central Committee on Research Involving Human Subjects, this study was exempt from ethical review in the Netherlands.

### Regional cancer networks

Cancer care in the Netherlands is organised into seven regional oncology networks,^13^ and all hospitals are affiliated with one of these seven networks. Hence, we assigned patients to the network corresponding to the hospital where their cancer was diagnosed. Regional oncology collaboration in the Netherlands has a long history, with multidisciplinary collaboration and regional guideline development dating back to the 1980s, initially organised around province-based regions. The current regional oncology networks evolved from this existing collaboration, with formal network structures developing gradually around 2013 and accelerating from 2015 onwards. To ensure anonymity and facilitate statistical comparison, networks were relabelled A through G in random order without geographic or alphabetical meaning. In the Supplementary Material, we show the hospitals per network, but at random without a label (Supplementary Table S1, available at https://doi.org/10.1016/j.esmorw.2026.100722).^13^

### Definitions

*De novo* MBC was defined as metastatic disease at the time of diagnosis of the primary tumour or within 3 months thereafter. HER2 positivity was defined according to national guidelines as immunohistochemistry 3+ or *in situ* hybridisation amplification of the primary tumour.^15^ Hormone receptor positivity was defined as nuclear staining in ≥10% of tumour cells in the primary tumour for oestrogen and/or progesterone receptors.^16^

Socioeconomic status (SES) was derived from Statistics Netherlands and based on the median disposable household income associated with the patient’s residential 4-digit postal code at the time of diagnosis, adjusted for household size. Twelve categories were collapsed into three groups (low, middle, and high). SES reflects area-level rather than individual income.

First-line treatment choice was classified as: (i) HER2-targeted therapy with or without chemotherapy or endocrine therapy, (ii) chemotherapy without HER2-targeted therapy, (iii) endocrine therapy without HER2-targeted therapy, and (iv) best supportive care only. Treatments initiated within 90 days were considered part of the initial treatment phase.

### Statistical analyses

Patient and tumour characteristics were summarised for the total cohort and by region (A-G). Variables included age at diagnosis [<50, 50-74, and ≥75 years, and median age with interquartile range (IQR)], sex (male and female), SES (low, middle, high, and unknown), clinical T-stage (cT0, cT1-2, cT3-4, and cTx), clinical N-stage (cN0, cN1-3, and cNx), histology (ductal, lobular, and other), grade of the primary tumour (1-2, 3, and unknown), hormone receptor status of the primary tumour (positive, negative, and unknown), number of organs involved (single and multiple), and initial site of metastatic disease [bone only, soft tissue without visceral or central nervous system (CNS), visceral without CNS, CNS, and unknown].^17^^,^^18^ The follow-up time was estimated using the reverse Kaplan–Meier method.

Differences across regions were assessed using the chi-square test for categorical variables and the Kruskal–Wallis test for continuous variables (median age). Missing data were handled using multiple imputation by chained equations with fully conditional specification in R (version 4.1.3; R Foundation for Statistical Computing, Vienna, Austria) (mice package). Polytomous logistic regression was used as the imputation method for all missing data. The predictor matrix included all study covariates. Twenty imputed datasets were generated (m = 20, maxit = 10).^19^

The primary endpoint was first-line treatment choice. Regional differences were first examined using unadjusted frequencies and compared across regions with the chi-square test. Subsequently, regional variation was evaluated using inverse probability of treatment weighting (IPTW), with region defined as the exposure. Covariates for IPTW were selected a priori based on clinical relevance and previous literature.^20^, ^21^, ^22^ These included incidence period, age group, SES, grade, hormone receptor status, number of organs involved, and initial site of metastatic disease. Covariate balance across the seven regions was evaluated using standardised mean differences (SMDs). An SMD <0.10 was considered acceptable. If residual imbalance remained after initial weighting, we evaluated interaction terms between selected baseline characteristics and incorporated those that improved covariate balance. The final multinomial model, including the interaction terms that yielded optimal balance, was used to generate the stabilised IPTW weights. IPTW-adjusted Poisson regression models with robust variance were then fitted to directly estimate risk ratios (RRs) and 95% confidence intervals (CIs). The multivariable Poisson regression model included region and additionally included the same covariates as a doubly robust specification. The region with the largest patient population served as the reference category. All *P* values were derived from Wald tests within the regression models. To quantify the absolute magnitude of regional differences, absolute risk differences with 95% CIs were additionally estimated using a linear probability model with an identity link function within the same IPTW-weighted survey design framework. A log link function was specified, enabling direct estimation of RRs.

The secondary endpoint was OS, defined from diagnosis to death or last follow-up, whichever occurred first.^23^ Both unadjusted and IPTW-adjusted Kaplan–Meier curves were generated to visualise OS by region. Median OS was estimated using the Kaplan–Meier method, and *P* values for survival differences were obtained from the log-rank test. Regional OS differences were further assessed using Cox proportional hazards regression with the same IPTW. Model one adjusted for patient and tumour characteristics (age, SES, grade, hormone receptor status, number of organs, and metastatic site). Model two additionally adjusted for first-line treatment choice. The region with the largest patient population served as the reference category in both Cox models.

All statistical analyses were performed in R. Figures were generated in R. Two-sided *P* values <0.05 were considered statistically significant, and *P* values <0.10 indicated a trend. This study followed the European Society for Medical Oncology (ESMO) Guidance for Reporting Oncology Real-World Evidence.^24^

## Results

**Figure 1:**
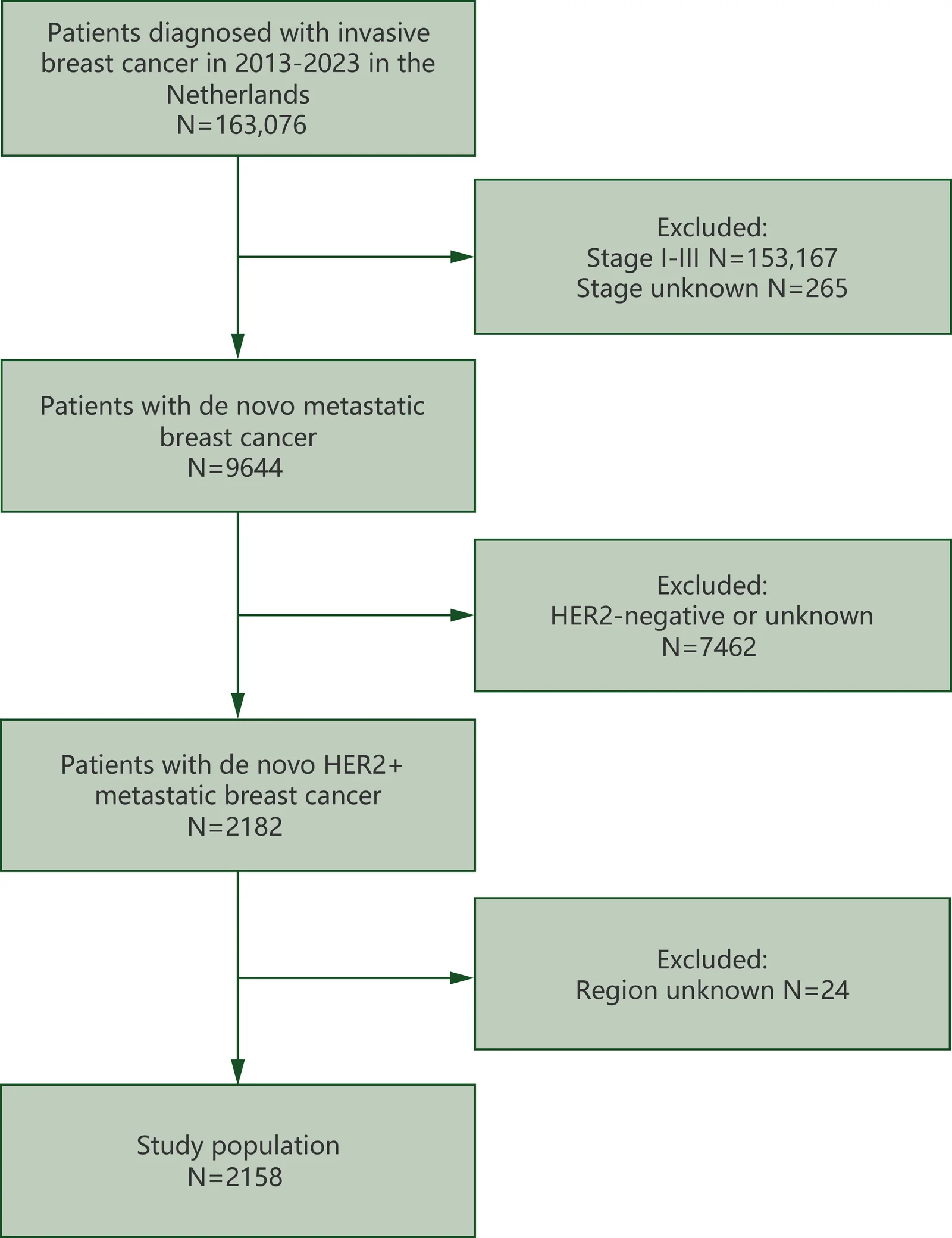
**Study flow chart.** HER2+, human epidermal growth factor receptor 2 positive.

Between 2013 and 2023, 163 076 patients were diagnosed with invasive breast cancer in the Netherlands, of whom 9644 (6%) presented with *de novo* MBC (Figure 1). Among these, 2182 (23%) had *de novo* HER2-positive MBC. After excluding 24 patients with missing information on the region of diagnosis, 2158 patients were included in the analysis. The median follow-up duration was 56.3 months (IQR 29.0-89.0).

### Baseline characteristics

The median age at diagnosis was 58 years (IQR 48-70). Of the patients, 30% were younger than 50 years, and 17% were aged 75 years or older (Table 1). Most patients had ductal histology (91%), grade 3 tumours (51%), and hormone receptor-positive disease (58%). Nearly half of the cohort had a high SES (45%), and 54% presented with metastases confined to a single organ. The most common sites of initial metastases were visceral (58%), bone only (24%), and soft tissue (14%).

**Table 1:** Baseline characteristics of patients with *de novo* HER2-positive MBC in the Netherlands in 2013-2023

Characteristics	Total	Region							
	*N* = 2158	A *N* = 463	B *N* = 252	C *N* = 342	D *N* = 256	E *N* = 196	F *N* = 233	G *N* = 416	*P* value
Incidence period									0.64
2019-2023	1107 (51)	237 (51)	121 (48)	171 (50)	135 (53)	112 (57)	120 (52)	211 (51)	
2013-2018	1075 (49)	226 (49)	131 (52)	171 (50)	121 (47)	84 (43)	113 (49)	205 (49)	
Age at diagnosis (years)									0.14
<50	645 (30)	121 (26)	81 (32)	119 (35)	83 (32)	61 (31)	60 (26)	120 (29)	
50-74	1144 (53)	256 (57)	133 (53)	176 (52)	127 (50)	104 (53)	121 (52)	218 (52)	
≥75	369 (17)	77 (17)	38 (15)	47 (14)	46 (18)	31 (16)	52 (22)	78 (19)	
Sex									0.59
Female	2141 (99)	458 (99)	251 (99)	340 (99)	253 (99)	193 (99)	231 (99)	415 (99)	
Male	17 (1)	5 (1)	1 (1)	2 (1)	3 (1)	3 (1)	2 (1)	1 (1)	
Socioeconomic status									<0.001
High	691 (32)	111 (24)	115 (46)	129 (38)	69 (27)	74 (38)	53 (23)	140 (34)	
Middle	787 (37)	192 (42)	78 (31)	115 (34)	106 (42)	48 (25)	106 (46)	142 (34)	
Low	661 (31)	151 (33)	57 (23)	96 (28)	80 (31)	73 (37)	72 (31)	132 (32)	
Unknown	*n* = 19	*n* = 9	*n* = 2	*n* = 2	*n* = 1	*n* = 1	*n* = 2	*n* = 2	
Clinical T-stage									0.30
cT0	33 (2)	12 (3)	3 (1)	5 (2)	2 (1)	4 (2)	4 (2)	3 (1)	
cT1-2	996 (48)	189 (55)	117 (48)	169 (51)	112 (45)	99 (54)	106 (47)	204 (51)	
cT3-4	1052 (51)	245 (71)	123 (51)	156 (47)	133 (54)	82 (44)	117 (52)	196 (49)	
cTx	*n* = 77	*n* = 17	*n* = 9	*n* = 12	*n* = 9	*n* = 11	*n* = 6	*n* = 13	
Clinical N-stage									0.50
cN0	247 (12)	62 (14)	20 (8)	40 (12)	26 (10)	28 (15)	27 (12)	44 (11)	
cN1-3	1864 (88)	392 (86)	227 (92)	291 (88)	227 (90)	162 (85)	201 (88)	364 (89)	
cNx	*n* = 47	*n* = 9	*n* = 5	*n* = 11	*n* = 3	*n* = 6	*n* = 5	*n* = 8	
Histology									0.66
Ductal	1961 (91)	415 (90)	230 (91)	315 (92)	238 (93)	177 (90)	213 (91)	373 (90)	
Lobular	158 (7)	40 (9)	20 (8)	23 (7)	15 (6)	13 (7)	14 (6)	33 (8)	
Other	39 (2)	8 (2)	2 (1)	4 (1)	3 (1)	6 (3)	6 (3)	10 (2)	
Grade^a^									<0.001
1-2	725 (49)	120 (40)	74 (49)	137 (54)	90 (54)	57 (37)	107 (54)	140 (52)	
3	766 (51)	178 (60)	77 (51)	116 (46)	78 (46)	97 (63)	90 (46)	130 (48)	
Unknown	*n* = 667	*n* = 165	*n* = 101	*n* = 89	*n* = 88	*n* = 42	*n* = 36	*n* = 146	
Hormone receptor^a^									0.50
Positive	1238 (58)	272 (59)	142 (57)	187 (55)	161 (63)	110 (57)	138 (59)	228 (55)	
Negative	910 (42)	190 (41)	108 (43)	152 (45)	94 (37)	84 (43)	95 (41)	187 (45)	
Unknown	*n* = 10	*n* = 1	*n* = 2	*n* = 3	*n* = 1	*n* = 2	*n* = 0	*n* = 1	
Number of organs involved									0.03
Single organ	1156 (54)	234 (51)	139 (55)	208 (61)	132 (52)	93 (47)	118 (51)	232 (56)	
Multiple organs	1002 (46)	229 (50)	113 (45)	134 (39)	124 (48)	103 (53)	115 (49)	184 (44)	
Initial metastatic site									0.55
Bone only	511 (24)	110 (24)	62 (25)	86 (25)	60 (23)	39 (20)	46 (20)	108 (26)	
Soft tissue^b^	309 (14)	59 (13)	37 (15)	58 (17)	33 (13)	24 (12)	38 (16)	60 (14)	
Visceral^c^	1256 (58)	269 (58)	144 (57)	188 (55)	152 (59)	124 (63)	141 (61)	238 (57)	
CNS^d^	82 (4)	25 (5)	9 (4)	10 (3)	11 (4)	9 (5)	8 (3)	10 (2)	

Values are *n* (%). Percentages may not add up to or exceed 100% because of rounding. Unknown data were not included in the percentages.

CNS, central nervous system, HER2, human epidermal growth factor receptor 2, MBC, metastatic breast cancer; N-stage, nodal stage, T-stage, tumour stage.

a Of the primary tumour.

b Lymph nodes and skin, excluding visceral or CNS.

c Liver, lung, pancreas, heart, pleura, peritoneum, gastrointestinal tract, kidney, endocrine glands, uterus, and ovaries, excluding CNS.

d Brain, meninges, and eye.

Across the seven regional cancer networks (A to G), SES and grade differed significantly (both *P* < 0.001). The proportion of high SES ranged from 24% in region A to 46% in region B, while grade 3 tumours ranged from 46% in regions C, D, and F to 63% in region E. The number of metastatic organs also showed a statistically significant regional difference (*P* = 0.03), with single-organ involvement ranging from 47% in region E to 61% in region C. Age distribution (*P* = 0.14), hormone receptor status (*P* = 0.50), and initial metastatic site (*P* = 0.20) were comparable across regions.

Patterns of missing data and the distribution of key baseline variables before and after multiple imputation are presented in Supplementary Table S2, available at https://doi.org/10.1016/j.esmorw.2026.100722. Before applying IPTW, several covariates, including diagnosis period, age, SES, grade, hormone receptor status, and number of organs involved, showed moderate imbalance (SMD >0.1; Supplementary Table S3a, available at https://doi.org/10.1016/j.esmorw.2026.100722). After IPTW, all variables achieved adequate balance, with SMD <0.1 (Supplementary Table S3b, available at https://doi.org/10.1016/j.esmorw.2026.100722).

### First-line treatment choice

**Figure 2:**
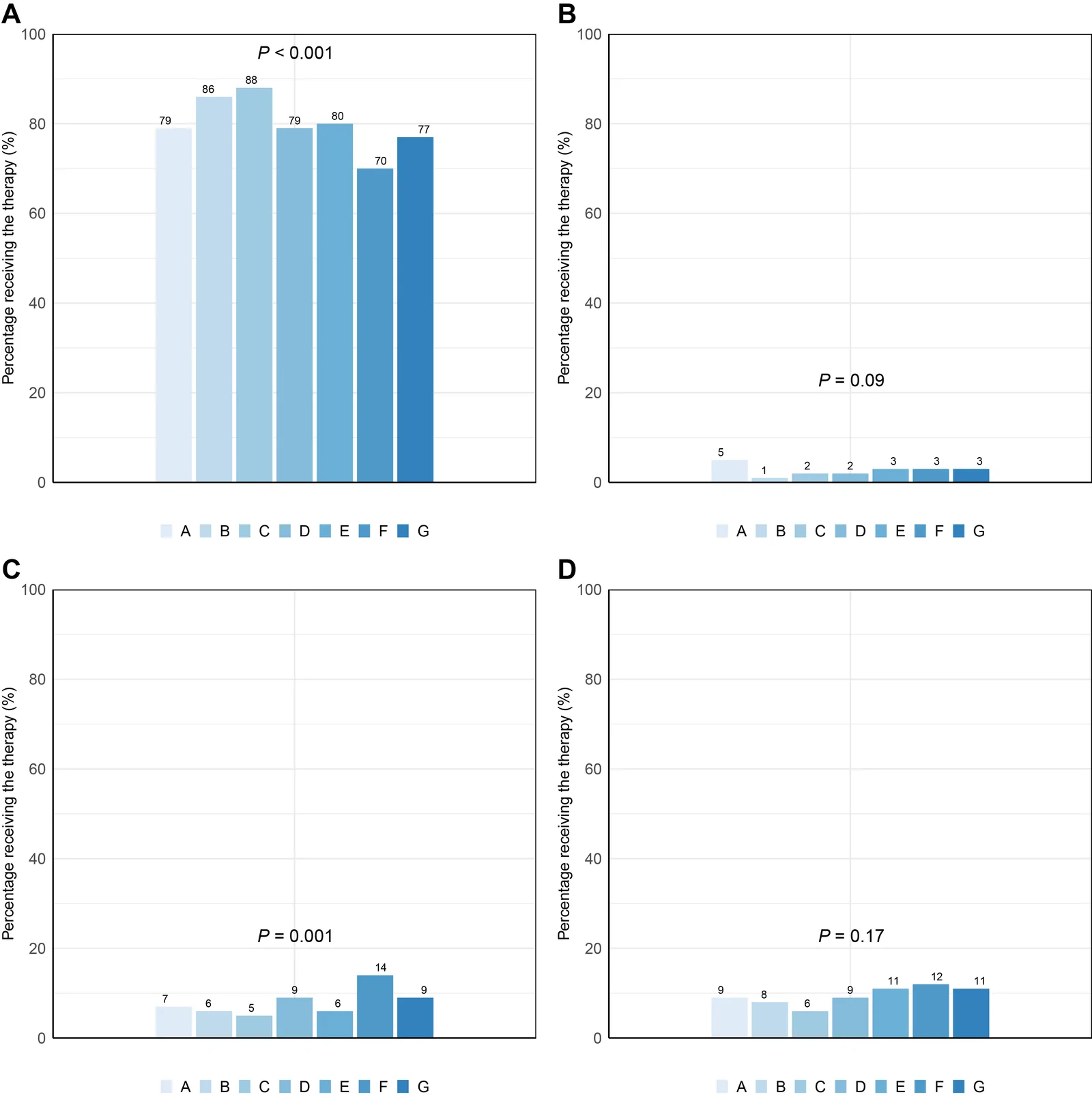
**First-line treatment choice of patients with *de novo* HER2-positive MBC in the Netherlands in 2013-2023.** (A) HER2-targeted therapy with or without chemotherapy or endocrine therapy, (B) chemotherapy without HER2-targeted therapy, (C) endocrine therapy without HER2-targeted therapy, and (D) best supportive care only. Percentages may not add up to or exceed 100% because of rounding. HER2, human epidermal growth factor receptor 2; MBC, metastatic breast cancer.

Among all 2158 patients, 1727 (80%) received first-line HER2-targeted therapy as part of their first-line treatment choice (Figure 2A). The proportion of patients receiving HER2-targeted therapy differed significantly between regions (*P* < 0.001), ranging from 70% in region F to 88% in region C. The use of endocrine therapy without HER2-targeted agents was relatively limited overall but varied significantly across regions (5%-14%, *P* < 0.001; Figure 2C). The proportions of patients treated with chemotherapy without HER2-targeted agents or best supportive care only were comparable across regions (1%-5%, *P* = 0.09 and 6%-12%, *P* = 0.17, respectively; Figure 2B and D).

After IPTW adjustment, patients in region C were statistically significantly more likely to receive HER2-targeted therapy than those in region A (RR 1.09, 95% CI 1.02-1.16, *P* = 0.01) (Table 2), although the relative difference was small. Use of HER2-targeted therapy was similar for regions B, D, E, F, and G when compared with region A (*P* ≥0.05).

**Table 2:** Regional variation in the use of HER2-targeted therapy among patients with *de novo* HER2-positive MBC (2013-2023): IPTW-based absolute risk differences and IPTW-adjusted Poisson regression

Region	Absolute risk difference (%, 95% CI)	Risk ratios^a^ (95% CI), *P* value
A	—	Ref.
B	5.8 (−0.2 to 11.8)	1.07 (1.00-1.15), 0.06
C	7.3 (2.1 to 12.6)	1.09 (1.02-1.16), 0.01
D	−0.9 (−6.8 to 5.1)	0.99 (0.92-1.07), 0.77
E	2.3 (−3.9 to 8.5)	1.03 (0.95-1.11), 0.48
F	−4.7 (−11.0 to 1.6)	0.94 (0.87-1.02), 0.15
G	−2.4 (−7.7 to 2.8)	0.97 (0.91-1.04), 0.35

CI, confidence interval; HER2, human epidermal growth factor receptor 2; IPTW, inverse probability of treatment weighting; MBC, metastatic breast cancer; Ref., reference.

a IPTW-adjusted model including region and baseline covariates (age, socioeconomic status, grade, receptor status, number of organs involved, and metastatic site).

### Overall survival

The overall median OS was 51.3 months (95% CI 47.6-56.3), ranging from 42.8 months (95% CI 36.2-51.8) in region A to 74.9 months (95% CI 49.9-not reached) in region B (Supplementary Figure S1, available at https://doi.org/10.1016/j.esmorw.2026.100722).

**Figure 3:**
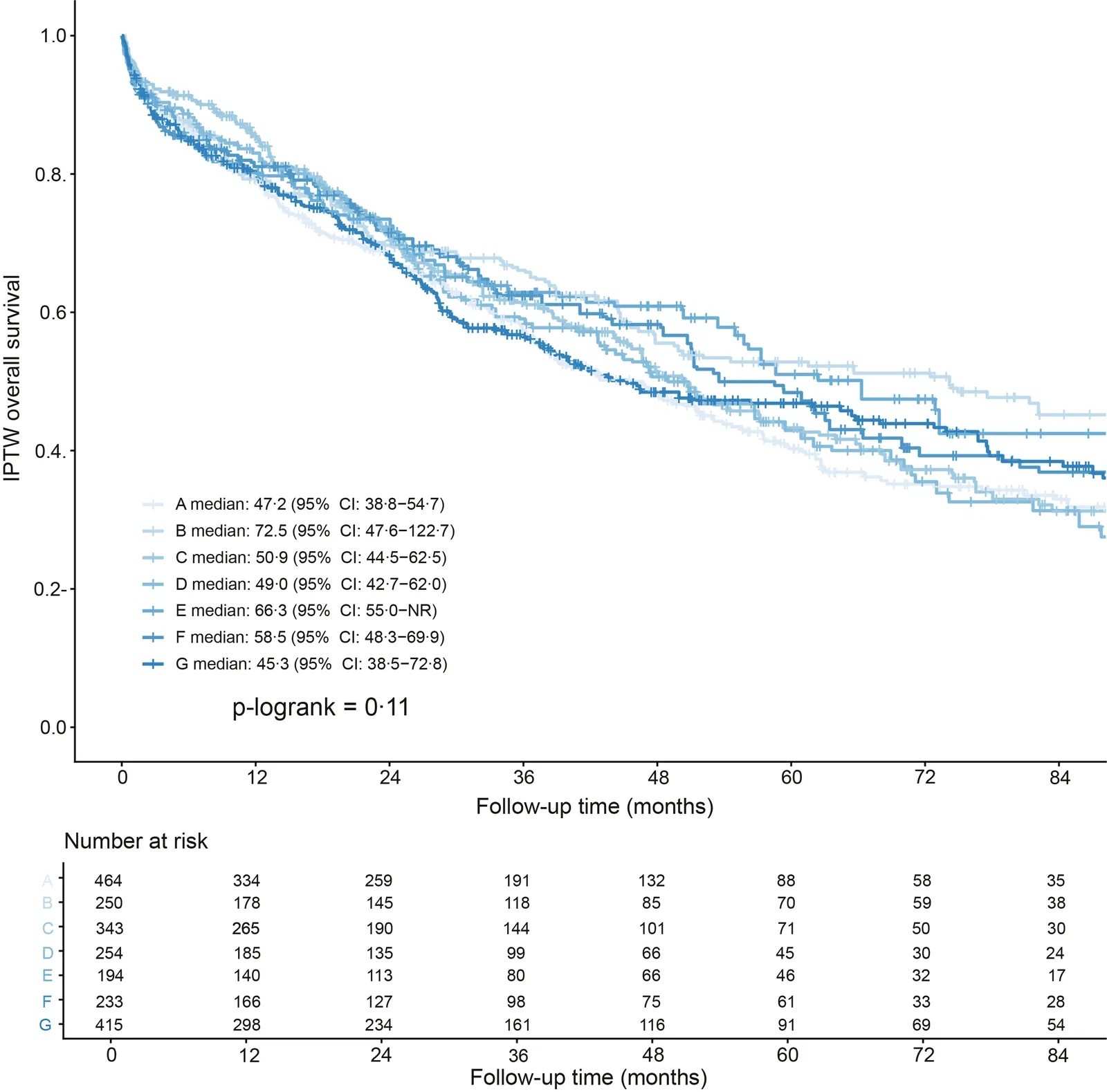
**IPTW-adjusted Kaplan–Meier curves of OS of patients systemically treated for *de novo* HER2-positive MBC by region in the Netherlands in 2013-2023.** CI, confidence interval; HER2, human epidermal growth factor receptor 2; IPTW, inverse probability of treatment weighting; MBC, metastatic breast cancer; OS, overall survival.

After IPTW, survival curves showed substantial overlap, and the log-rank test did not indicate a statistically significant difference (log-rank *P* = 0.11) (Figure 3).

After adjustment for patient and tumour characteristics (i.e. IPTW-adjusted Cox regression model 1), patients from region B [hazard ratio (HR) 0.69, 95% CI 0.54-0.89, *P* = 0.005] and region E (HR 0.74, 95% CI 0.56-0.97, *P* = 0.03) showed better OS compared with those in region A (Table 3). For all other regions, no statistically significant survival differences were observed compared with region A. After further adjustment for first-line treatment choice (i.e. model 2), the results hardly changed (region B: HR 0.77, *P* = 0.06; region E: HR 0.77, *P* = 0.07). This indicates that first-line treatment choice was not accountable for the observed borderline significant survival differences.

**Table 3:** Regional differences in OS among patients with *de novo* HER2-positive MBC (2013-2023): IPTW-adjusted Cox regression

Region	Model 1^a^	Model 2^b^
A	Ref.	Ref.
B	0.69 (0.54-0.89), 0.005	0.77 (0.59-1.01), 0.06
C	0.82 (0.66-1.01), 0.06	0.92 (0.74-1.15), 0.46
D	0.88 (0.70-1.12), 0.30	0.89 (0.69-1.15), 0.36
E	0.74 (0.56-0.97), 0.03	0.77 (0.59-1.02), 0.07
F	0.81 (0.63-1.04), 0.10	0.83 (0.64-1.07), 0.15
G	0.91 (0.75-1.11), 0.35	0.91 (0.74-1.12), 0.39

Values are HR (95% CI), *P* value.

CI, confidence interval; HER2, human epidermal growth factor receptor 2; HR, hazard ratio; IPTW, inverse probability of treatment weighting; MBC, metastatic breast cancer; OS, overall survival; Ref., reference.

a IPTW-adjusted Cox regression including region, age, SES, grade, hormone receptor status, number of metastatic organs, and initial metastatic site.

b IPTW-adjusted Cox regression is as in model 1, additionally adjusted for first-line treatment.

## Discussion

Our nationwide population-based study found only modest regional variation in the use of first-line HER2-targeted therapy and OS among patients with *de novo* HER2-positive MBC in the Netherlands. Although crude differences in treatment and OS were observed across the seven oncology networks, these differences diminished mainly after accounting for baseline patient characteristics through IPTW weighting. The results suggest that apparent regional differences are primarily driven by case mix (i.e. differences in patient and tumour characteristics) rather than inequities in treatment access or clinical care. Further adjustment for first-line treatment attenuated the HRs only modestly; however, as treatment choice is itself influenced by baseline patient and tumour characteristics, this contribution cannot be fully disentangled from the underlying case mix.

Previous studies have reported regional variation in access to cancer medicines across European countries.^25^ In England, registry-based analyses showed significant regional variation in the use of systemic anticancer therapy and survival outcomes among patients with surgically treated early breast cancer, despite the presence of a unified national health system and national treatment guidelines.^26^ Similarly, Dutch population-based studies have identified regional differences in the multidisciplinary management of breast cancer.^27^ However, the magnitude of these variations was limited, and overall adherence to national guidelines remained high. Given these observations, understanding whether regional variation also exists within the standardised Dutch oncology network is essential to evaluate the effectiveness of national coordination in ensuring equitable access and outcomes for patients with *de novo* HER2-positive MBC.

The Netherlands provides a unique setting to study regional differences in real-world cancer care. Cancer management is organised within national oncology networks that integrate general, teaching, and academic hospitals through shared treatment pathways and multidisciplinary tumour boards. These networks, coordinated through organisations such as the National Breast Cancer Organisation of the Netherlands, aim to promote uniform access to systemic therapy and centralise decision making for complex cases.^28^ Many hospitals, particularly academic centres, are also members of the Organisation of European Cancer Institutes, facilitating collaboration, benchmarking, and exchange of best practices.^29^ Within these networks, structured tumour boards and peer-review processes support the application of evidence-based care and help integrate innovations efficiently into practice.^27^ Our observation that no large regional differences in first-line HER2-targeted therapy use were seen, together with only small regional variations in OS that were largely attributable to baseline patient characteristics, suggests that the coordinated national approach to cancer care has been largely successful. High adherence to national guidelines, early nationwide adoption of HER2-targeted agents, and continuous feedback through the NCR likely contribute to these consistent outcomes. As treatment options for MBC continue to expand, strengthening mechanisms for multidisciplinary collaboration, guideline adherence, and equitable access will remain essential to sustain the success of this system.

Our findings suggest that a nationally coordinated oncology network may contribute to broadly uniform treatment delivery and survival outcomes for patients with *de novo* HER2-positive MBC, although a causal interpretation cannot be drawn from the current observational study design. These findings reinforce the value of centralised guideline development, structured multidisciplinary collaboration, and continuous feedback through national cancer registries. For countries aiming to achieve equity in cancer care, the Dutch model provides a strong example of how organisational design can translate into uniform access and consistent survival outcomes. Future research should evaluate whether similar levels of uniformity are maintained as novel agents, such as antibody-drug conjugates and tyrosine kinase inhibitors, become integrated into clinical practice. Linking registry data with information on comorbidity, performance status, and treatment sequencing will further clarify potential sources of residual variation and help sustain equitable, evidence-based care across regions.

This nationwide, population-based study has several strengths. The large real-world cohort enhances the reliability and clinical relevance of the findings. By including all regions of the Netherlands, the study reflects real-world variation and supports generalisability. Use of comprehensive cancer registry data ensured consistent reporting of patient and tumour characteristics and first-line treatment patterns. Some limitations should also be noted. Although treatment differences may sometimes be greater between individual hospitals than between regions, coordinated regional cancer networks in the Netherlands likely minimise unwarranted hospital-level variation, and hospital-level analyses would not have been feasible due to small patient numbers per hospital. Although IPTW achieved an excellent balance across all observed covariates, residual confounding cannot be completely ruled out because some clinically relevant factors were not available in the dataset. First, information on comorbidities, frailty, and performance status was lacking. These factors influence both the likelihood of receiving HER2-targeted therapy and overall prognosis and may vary across regions due to differences in population health or lifestyle. For example, heart failure or severe lung disease can preclude the safe use of HER2-targeted agents. Second, SES was measured at the postal-code level rather than at the individual level, which could introduce misclassification.^30^ Third, data on subsequent systemic therapies were unavailable. Regional variation in access to later-line HER2-targeted agents, such as trastuzumab emtansine (T-DM1), tucatinib-based regimens, or trastuzumab deruxtecan, may affect survival beyond first-line treatment. Finally, hospital type and local referral patterns were not captured and could reflect structural differences across networks. Despite these limitations, the adjusted analyses suggest that most of the crude regional variation is attributable to case mix rather than to differences in the way care was delivered. As this was an observational study with prespecified regions, no formal a priori power calculation was performed. Based on ESMO-MCBS v2.0 Form 2a criteria for settings with median OS ≥36 months, an HR >0.75 or absolute OS gain <6 months is not considered clinically meaningful.^31^ The observed regional differences in OS did not reach this threshold after adjustment for first-line treatment. For HER2-targeted therapy use, the observed RRs were similarly not clinically meaningful.

### Conclusion

In conclusion, our findings indicate that the delivery of first-line HER2-targeted therapy for *de novo* HER2-positive MBC is broadly consistent across Dutch oncology networks, with no meaningful regional difference after adjustment. The difference in observed survival appears attributable mainly to the case mix rather than to the differential quality of care. These results support the notion of equitable access to systemic therapy within the Netherlands and highlight the strength of a coordinated, guideline-driven oncology system.
