# Supplementary material for: Treatment and survival of de novo HER2-positive metastatic breast cancer across regional cancer networks in the Netherlands: a cohort study
Source: ESMO Real World Data Digit Oncol. 2026 Aug 24;13:100722. doi: 10.1016/j.esmorw.2026.100722 (PMC13542605; doi:10.1016/j.esmorw.2026.100722)
Supplement: Supplementary file 1 [file mmc1.docx]

**Supplementary table for “Treatment and survival of *de Novo* HER2-positive metastatic breast cancer across regional cancer networks in the Netherlands: a cohort study”**

**Supplementary Table 1. Oncology networks and hospitals in the Netherlands**

| **Oncology network regions** | **Hospitals** |
| --- | --- |
| Oncologienetwerk North | Martini Ziekenhuis  Ommelander Ziekenhuis Groningen (OZG)  Treant zorggroep locatie Refaja  Universitair Medisch Centrum Groningen (UMCG)  Wilhelmina Ziekenhuis Assen  Antonius Ziekenhuis Sneek  Heelkunde Friesland  Medisch Centrum Leeuwarden (MCL)  Radiotherapeutisch Instituut Friesland (RIF)  Ziekenhuis Nij Smellinghe  Ziekenhuis Tjongerschans  Deventer Ziekenhuis  Gelre Zutphen  Isala  Stichting Gelre Ziekenhuizen |
| Oncologienetwerk Midden-Nederland | Alexander Monro Ziekenhuis  Diakonessenhuis, locatie Utrecht  Diakonessenhuis, locatie Zeist  Meander Medisch Centrum  St. Antonius Ziekenhuis  UMC Utrecht locatie WKZ  UMC Utrecht (UMCU)  Ziekenhuis Rivierenland (ZRT)  Tergooi locatie Hilversum |
| Oncologienetwerk West-Nederland | Alrijne Ziekenhuis Leiderdorp/Leiden/Alphen aan den Rijn (zorggroep)  Groene Hart Ziekenhuis  HagaZiekenhuis, loc. Leyweg  HMC (Haaglanden Medisch Centrum)  HMC Antoniushove  Leids Universitair Medisch Centrum (LUMC)  Reinier de Graaf |
| Oncologienetwerk Oost-Nederland | Medisch Spectrum Twente  Saxenburg Medisch Centrum  Streekziekenhuis Koningin Beatrix Winterswijk  ZGT Hengelo  Ziekenhuisgroep Twente (ZGT)  Ziekenhuis Gelderse Vallei  Jeroen Bosch Ziekenhuis  Canisius-Wilhelmina Ziekenhuis  Maasziekenhuis Pantein  Radboudumc  Rijnstate Arnhem  Slingeland Ziekenhuis  Ziekenhuis Bernhoven B.V. |
| Oncologienetwerk Noord-Holland-Flevoland | Amsterdam UMC - Locatie AMC  Amsterdam UMC - locatie VUmc  Antoni van Leeuwenhoek ziekenhuis / NKI  BovenIJ ziekenhuis  Dijklander Ziekenhuis (Hoorn, Purmerend)  Flevoziekenhuis  Noordwest Ziekenhuisgroep Alkmaar  Noordwest Ziekenhuisgroep locatie Den Helder  OLVG, locatie Oost  Rode Kruis Ziekenhuis  Spaarne Gasthuis  Spaarne Gasthuis. loc. Haarlem-Zuid  Zaans Medisch Centrum  Ziekenhuis Amstelland  Ziekenhuis St. Jansdal |
| Oncologienetwerk Zuidoost-Nederland | Anna Ziekenhuis  Catharina Ziekenhuis  Elkerliek Ziekenhuis  Laurentius Ziekenhuis  Maastricht UMC+  Máxima Medisch Centrum Veldhoven  SJG Weert (St. Jans Gasthuis)  VieCuri Medisch Centrum  Zuyderland Medisch Centrum  Zuyderland, locatie Heerlen  Zuyderland, locatie Sittard-Geleen |
| Oncologienetwerk Zuidwest-Nederland (EMBRAZE en CONCORD) | Albert Schweitzer Ziekenhuis  Franciscus Gasthuis & Vlietland  Franciscus Vlietland & Gasthuis  IJsselland Ziekenhuis  Ikazia Ziekenhuis  Maasstad Ziekenhuis  Spijkenisse Medisch Centrum  Van Weel-Bethesda Ziekenhuis  Rivas Zorggroep Beatrix Ziekenhuis  Erasmus MC  Erasmus MC Kanker Instituut  Admiraal De Ruyter Ziekenhuis  Amphia Ziekenhuis  Amphia Ziekenhuis locatie Langendijk  Bravis Ziekenhuis (Bergen op Zoom/Roosendaal)  ETZ Elisabeth  ZorgSaam zorggroep Zeeuws-Vlaanderen |

**Supplementary Table 2. Key baseline characteristics before and after multiple imputation, N (%)**

|  | **Before imputation** | | **After imputation** |
| --- | --- | --- | --- |
|  |  |  |  |
| **Characteristics** | N=2158 | | |
| **Socioeconomic status** |  | | |
| High | 691 (32) | 697 (32) | |
| Middle | 787 (37) | 793 (37) | |
| Low | 661 (31) | 668 (32) | |
| Unknown | N=19 | N=0 | |
| **Grade*** |  | |  |
| 1-2 | 725 (49) | | 1051 (49) |
| 3 | 766 (51) | | 1107 (51) |
| Unknown | N=667 | | N=0 |
| **Hormone receptor status*** |  | |  |
| Positive | 1238 (58) | | 1243 (58) |
| Negative | 910 (42) | | 915 (42) |
| Unknown | N=10 | | N=0 |

N = number of patients. *Of the primary tumour. Percentages may not add up to or exceed 100% because of rounding.

**Supplementary Table 3. Baseline characteristics of the patients before and after the IPTW adjustment, with imputed data, N (%)**

**a. before IPTW**

| **Variable** |  | | **Before IPTW** | | | | | | |
| --- | --- | --- | --- | --- | --- | --- | --- | --- | --- |
|  | **A**  **N=463** | **B**  **N=252** | | **C**  **N=342** | **D**  **N=256** | **E**  **N=196** | **F**  **N=233** | **G**  **N=416** | **SMD** |
| **Incidence period** |  |  | |  |  |  |  |  |  |
| 2019-2023 | 237 (51) | 121 (48) | | 171 (50) | 135 (53) | 112 (57) | 120 (52) | 211 (51) | -0·12 |
| 2013-2018 | 226 (49) | 131 (52) | | 171 (50) | 121 (47) | 84 (43) | 113 (49) | 205 (49) | 0·12 |
| **Age at diagnosis** |  |  | |  |  |  |  |  |  |
| < 50 years | 121 (26) | 81 (32) | | 119 (35) | 83 (32) | 61 (31) | 60 (26) | 120 (29) | 0·11 |
| 50-74 years | 256 (57) | 133 (53) | | 176 (52) | 127 (50) | 104 (53) | 121 (52) | 218 (52) | 0·08 |
| ≥ 75 years | 77 (17) | 38 (15) | | 47 (14) | 46 (18) | 31 (16) | 52 (22) | 78 (19) | 0·13 |
| **Socioeconomic status** |  |  | |  |  |  |  |  |  |
| High | 113 (24) | 117 (46) | | 130 (38) | 69 (27) | 74 (38) | 54 (23) | 140 (34) | 0·29 |
| Middle | 194 (42) | 78 (31) | | 115 (34) | 107 (42) | 49 (25) | 106 (46) | 144 (35) | -0·26 |
| Low | 156 (34) | 57 (23) | | 97 (28) | 80 (31) | 73 (37) | 73 (31) | 132 (32) | -0·19 |
| **Grade*** |  |  | |  |  |  |  |  |  |
| 1-2 | 193 (42) | 117 (46) | | 179 (52) | 135 (53) | 74 (38) | 125 (54) | 215 (52) | -0·21 |
| 3 | 270 (58) | 135 (54) | | 163 (48) | 121 (47) | 122 (62) | 108 (46) | 210 (48) | 0·21 |
| **Hormone receptor*** |  |  | |  |  |  |  |  |  |
| Positive | 273 (59) | 143 (57) | | 187 (55) | 162 (63) | 111 (57) | 138 (59) | 228 (55) | 0·12 |
| Negative | 190 (41) | 109 (43) | | 155 (45) | 94 (37) | 85 (43) | 95 (41) | 188 (45) | -0·12 |
| **Number of organs involved** |  |  | |  |  |  |  |  |  |
| Single organ | 234 (51) | 139 (55) | | 208 (61) | 132 (52) | 93 (47) | 118 (51) | 232 (56) | 0·15 |
| Multiple organs | 229 (50) | 113 (45) | | 134 (39) | 124 (48) | 103 (53) | 115 (49) | 184 (44) | -0·15 |
| **Initial metastatic site** |  |  | |  |  |  |  |  |  |
| Bone only | 110 (24) | 62 (25) | | 86 (25) | 60 (23) | 39 (20) | 46 (20) | 108 (26) | -0·10 |
| Soft tissue^1^ | 59 (13) | 37 (15) | | 58 (17) | 33 (13) | 24 (12) | 38 (16) | 60 (14) | 0.07 |
| Visceral^2^ | 269 (58) | 144 (57) | | 188 (55) | 152 (59) | 124 (63) | 141 (61) | 238 (57) | 0.10 |
| CNS^3^ | 25 (5) | 9 (4) | | 10 (3) | 11 (4) | 9 (5) | 8 (3) | 10 (2) | 0.08 |

CNS= central nervous system, HER2+= human epidermal growth factor receptor 2 positive, IPTW= inverse probability of treatment weighting, SMD= Standardized mean difference. Percentages may not add up to or exceed 100% because of rounding. *Of the primary tumour. ^1^Lymph nodes and skin, without visceral or CNS. ^2^Liver, lung, pancreas, heart, pleura, peritoneum, gastrointestinal track, kidney, endocrine glands, uterus and ovaries, without CNS. ^3^Brain, meninges, and eye.

**b. after IPTW**

| **Variable** | **After IPTW** | | | | | | | |
| --- | --- | --- | --- | --- | --- | --- | --- | --- |
|  | **A**  **N=464·3** | **B**  **N=250·4** | **C**  **N=343·2** | **D**  **N=253·7** | **E**  **N=194·3** | **F**  **N=233·3** | **G**  **N=415·0** | **SMD** |
| **Incidence period** |  |  |  |  |  |  |  |  |
| 2019-2023 | 233·2 (50) | 124·8 (50) | 173·8 (51) | 131·8 (52) | 100·2 (52) | 121·1 (52) | 212·7 (51) | -0·02 |
| 2013-2018 | 231·1 (50) | 125·6 (50) | 169.4 (49) | 121·9 (48) | 94·1 (48) | 112·2 (48) | 202·3 (49) | 0·02 |
| **Age at diagnosis** |  |  |  |  |  |  |  |  |
| < 50 years | 140·4 (30) | 74·8 (30) | 100·3 (30) | 77·6 (31) | 58·7 (30) | 68·7 (30) | 127·2 (31) | 0·01 |
| 50-74 years | 243·1 (52) | 133·5 (53) | 185.3 (54) | 134·0 (53) | 105·7 (54) | 125·9 (54) | 217·2 (52) | 0·03 |
| ≥ 75 years | 80·9 (17) | 42·1 (17) | 57·6 (17) | 42·1 (17) | 29·9 (15) | 38·7 (17) | 70·6 (17) | -0·03 |
| **Socioeconomic status** |  |  |  |  |  |  |  |  |
| High | 148·9 (32) | 81·6 (33) | 111·7 (33) | 83·4 (33) | 62·4 (32) | 75·0 (32) | 133·8 (32) | 0·01 |
| Middle | 171·6 (37) | 92·4 (37) | 126·7 (37) | 95·0 (37) | 71·2 (37) | 86·2 (37) | 152·3 (37) | 0·01 |
| Low | 143·8 (31) | 76·5 (31) | 104·8 (31) | 75·3 (30) | 60·7 (31) | 72·1 (31) | 128·9 (31) | -0·02 |
| **Grade*** |  |  |  |  |  |  |  |  |
| 1-2 | 223·6 (48) | 117·3 (47) | 166·6 (49) | 119·9 (47) | 91·1 (47) | 112·2 (48) | 202·6 (49) | -0·02 |
| 3 | 240·8 (52) | 133·1 (53) | 176·7 (51) | 133·8 (53) | 103·2 (53) | 121·2 (52) | 212·4 (51) | 0·02 |
| **Hormone receptor*** |  |  |  |  |  |  |  |  |
| Positive | 271·0 (58) | 145·2 (58) | 203·9 (59) | 143·1 (56) | 114·7 (59) | 136·4 (58) | 238·2 (57) | -0·04 |
| Negative | 193·3 (42) | 105·3 (42) | 139·3 (41) | 110·6 (44) | 79·6 (41) | 97·0 (42) | 176·8 (43) | 0·04 |
| **Number of organs involved** |  |  |  |  |  |  |  |  |
| Single organ | 249·5 (54) | 132·7 (53) | 181·6 (53) | 137·2 (54) | 103·0 (53) | 123·2 (53) | 225·2 (54) | 0·02 |
| Multiple organs | 214·8 (46) | 117·7 (47) | 161·6 (47) | 116·5 (46) | 91·3 (47) | 110·1 (47) | 189·8 (46) | -0·02 |
| **Initial metastatic site** |  |  |  |  |  |  |  |  |
| Bone only | 109·7 (24) | 60·7 (24) | 81·8 (24) | 63·1 (25) | 45·7 (24) | 54·3 (23) | 100·6 (24) | 0·02 |
| Soft tissue^1^ | 67·8 (15) | 33·7 (13) | 48·5 (14) | 34·4 (14) | 26·2 (14) | 31·8 (14) | 60·9 (15) | -0·02 |
| Visceral^2^ | 269·0 (58) | 146·7 (59) | 199·5 (58) | 149·0 (59) | 115·2 (59) | 138·1 (59) | 238·2 (57) | 0·02 |
| CNS^3^ | 17·8 (4) | 9·4 (4) | 13·5 (4) | 7·2 (3) | 7·2 (4) | 9·2 (4) | 15·4 (4) | -0·05 |

CNS= central nervous system, HER2+= human epidermal growth factor receptor 2 positive, IPTW= inverse probability of treatment weighting, SMD= Standardized mean difference. Percentages may not add up to or exceed 100% because of rounding. *Of the primary tumour. ^1^Lymph nodes and skin, without visceral or CNS. ^2^Liver, lung, pancreas, heart, pleura, peritoneum, gastrointestinal track, kidney, endocrine glands, uterus and ovaries, without CNS. ^3^Brain, meninges, and eye.

**Supplementary Figure 1. Unadjusted Kaplan-Meier curves of overall survival of patients systemically treated for *de Novo* HER2+ metastatic breast cancer by region in the Netherlands in 2013-2023**

NR= not reached.
